# Transcriptome sequencing identified the ceRNA network associated with recurrent spontaneous abortion

**DOI:** 10.1186/s12920-021-01125-4

**Published:** 2021-11-23

**Authors:** Yong Huang, Jiayuan Hao, Yuan Liao, Lihua Zhou, Kaiju Wang, Hui Zou, Ying Hu, Juan Li

**Affiliations:** Department of Reproductive Medicine, The Second Affiliated Hospital of Hainan Medical University, No. 368, Yehai Avenue, Haikou, 570311 Hainan People’s Republic of China

**Keywords:** Recurrent spontaneous abortion, Transcriptome sequencing, lncRNA, ceRNA network

## Abstract

**Background:**

Recurrent spontaneous abortion (RSA) is one of the common complication of pregnancy, bringing heavy burden to the patients and their families. The study aimed to explore the lncRNA-miRNA-mRNA network associated with recurrent spontaneous abortion.

**Methods:**

By transcriptome sequencing, we detected differences in lncRNA, miRNA and mRNA expression in villus tissue samples collected from 3 patients with RSA and 3 normal abortion patients. Differentially expressed lncRNAs, miRNAs and genes (DELs, DEMs and DEGs, respectively) were identified, and Geno Ontology (GO) and Kyoto Encyclopedia of Genes and Genomes (KEGG) analyses were used to determine the functions of DELs and DEGs, which were analysed by Fisher’s test. We also observed the regulatory relationships between miRNA-mRNA and lncRNA-miRNA by Cytoscape 3.6.1.

**Results:**

The results showed that 1008 DELs (523 upregulated and 485 downregulated), 475 DEGs (201 upregulated and 274 downregulated) and 37 DEMs (15 upregulated and 22 downregulated) were identified. And we also constructed a novel lncRNA-related ceRNA network containing 31 lncRNAs, 1 miRNA (hsa-miR-210-5p) and 3 genes (NTNG2, GRIA1 and AQP1).

**Conclusions:**

lncRNA-related ceRNA network containing 31 lncRNAs, 1 miRNA (hsa-miR-210-5p) and 3 mRNAs (NTNG2, GRIA1 and AQP1) was constructed. The results may provide a basic theory for elucidating the mechanism underlying RSA.

**Supplementary Information:**

The online version contains supplementary material available at 10.1186/s12920-021-01125-4.

## Background

Recurrent spontaneous abortion (RSA), a complication of pregnancy, is defined as three or more consecutive spontaneous abortions with the same spouse, and gestational age < 20 weeks of spontaneous abortion [1]. The incidence rate of that was about 5%. To date, chromosomal abnormalities of parents and embryos, anatomical factors, thrombosis, immunological factors, endocrine factors and environmental factors have been reported to be correlated with the occurrence of RSA [2–5]. RSA seriously endangers women's reproductive health and causes great physical and mental pain for patients and their families. In recent years, new risk factors have been gradually recognized, but the aetiology of many patients with RSA remains unknown. At present, the commonly used treatment methods include immunotherapy, endocrine therapy and anticoagulant therapy. However, the effects of these treatments are not satisfactory. Therefore, it is of great significance to study the aetiology of RSA.

Noncoding RNAs play an important role in the almost all pathological or pathological processes, such as embryonic development, cell proliferation, differentiation, apoptosis, infection and immune response, including RSA [6, 7]. Long noncoding RNAs (lncRNAs), highly conserved noncoding RNAs, have also been found to be involved in RSA related studies [8–10]. Gu et al. observed [8] that polymorphisms in lncRNA HULC may be related to the susceptibility to RSA in the Southern Chinese population. Xuan et al. also found that the lncRNA MALAT1 rs619586 G mutation reduced the risk of RSA [9]. Che et al. found that lncRNA CCAT2 rs619586 G mutation may have a potential protective effect and reduce the risk of RSA in southern China [10]. The results described above indicated that lncRNAs played a role in RSA. Furthermore, miRNAs have also been found to be indispensable for the pathogenesis of RSA [11, 12]. By assessing the influence of USP25 on trophoblasts, Ding et al. found that USP25 expression was negatively regulated by miR-27a-3p, and this effect contributed to the pathogenesis of RSA by suppressing the migration and invasion of trophoblasts [11]. It was observed that the upregulation of miR-365 expression may promote the occurrence of RSA by reducing the expression of SGK1, suggesting that miR-365 may be used as a prognostic biomarker and therapeutic target for RSA reported by Zhao et al. [12]. As a new model of gene expression regulation, the large regulatory network of ceRNAs is helpful for exploring the gene function and regulatory mechanisms at a deeper level and for more thoroughly and comprehensively understanding many biological phenomena. However, so far, lncRNA-miRNA interactions and lncRNA-miRNA-mRNA networks have not been reported in RSA.

In the study, we constructed a lncRNA-associated ceRNA network to explore the pathogenesis of RSA in 3 patients with RSA and 3 normal abortion patients, providing a theoretical basis for the elucidation and treatment of RSA in the future.

## Material and methods

### Subjects

The villus tissue samples were collected from 3 patients with RSA and villus tissue samples from 3 normal abortion patients were served as controls. The fresh tissues were stored in liquid nitrogen tanks for subsequent use. The inclusion criteria for the RSA patients were as follows: (1) patients with RSA suffered three or more consecutive spontaneous abortions at a gestational age of < 20 weeks; (2) female patients with RSA who suffered primary abortion and had no previous history of live births; (3) RSA patients underwent routine examinations, including examination of maternal infection, chromosome aberration, endocrine dysfunction, anatomical factors and autoimmune diseases. Patients who did not meet these conditions were excluded. The controls had at least one childbirth and had no history of spontaneous abortion. Moreover, the controls had no pregnancy-related complications. All the subjects have signed an informed consent form. The study was approved by the Second Affiliated Hospital of Hainan Medical College (2018R005-F01).

### Transcriptome sequencing data analysis

Using the TRIzol Reagent (Thermo Fisher Science, USA), we extracted total RNA from the villus tissue samples. Subsequently, we measured the RNA concentration and purity. We performed lncRNA, miRNA and mRNA sequencing with the Illumina transcriptome chip. FastQC software and the R package (http://www.bioinformatics.babraham.ac.uk/projects/fastqc/) were used to evaluate the quality of the original sequencing data. Using the Trimgalore method (http://www.bioinformatics.babraham.ac.uk/projects/trim_Galore/), we filtered raw reads to obtain clean reads for subsequent analysis. Besides, all the data were processed by quantile normalization.

### Analysis of differentially expressed lncRNAs, miRNAs and genes

We used the Cuffdiff version 2.2.1 to identify differentially expressed lncRNAs, miRNAs and genes (DELs, DEMs and DEGs) in the villus tissue samples collected from 3 patients with RSA and 3 controls. *p* < 0:05 and |log_2_FC| > 1 were used as the screening criteria. We completed the heatmap analysis of DELs, DEMs and DEGs with the ComplexHeatmap in the R package.

### Functional analyses

In the present studie, Geno Ontology (GO) and Kyoto Encyclopedia of Genes and Genomes (KEGG) analyses were used to determine the functions of DELs and DEGs, which were analysed by Fisher’s test using the clusterProfiler version 2.2.1. Biological process (BP), cell composition (CC) and molecular function (MF) were included in GO annotation analysis. KEGG enrichment analysis mainly focused on the related signaling pathways. *p* < 0.05 was regarded statistically significant.

### Constructing the ceRNA network

We also observed the regulatory relationships between miRNA-mRNA and lncRNA-miRNA by Cytoscape version 3.6.1. The miRNA-mRNA-lncRNA network was constructed and visualized. According to the lncRNAs that directly interacted with mRNAs and regulated their activity as miRNA sponge, we analyze the data through the following three steps: (1) miRNAs targeted by DELs and the interaction between DELs and miRNAs were predicted using the LncTar software; (2) mRNAs targeted by DEMs and the interaction between DELs and miRNAs were predicted by the online tool (MiRDB, miRTarBase and Targetscan databases; (3) lncRNAs and miRNAs negatively regulated by mRNAs were integrated to construct a ceRNA network.

## Results

### Identification of differentially expressed lncRNAs, miRNAs and mRNAs

**Fig. 1:**
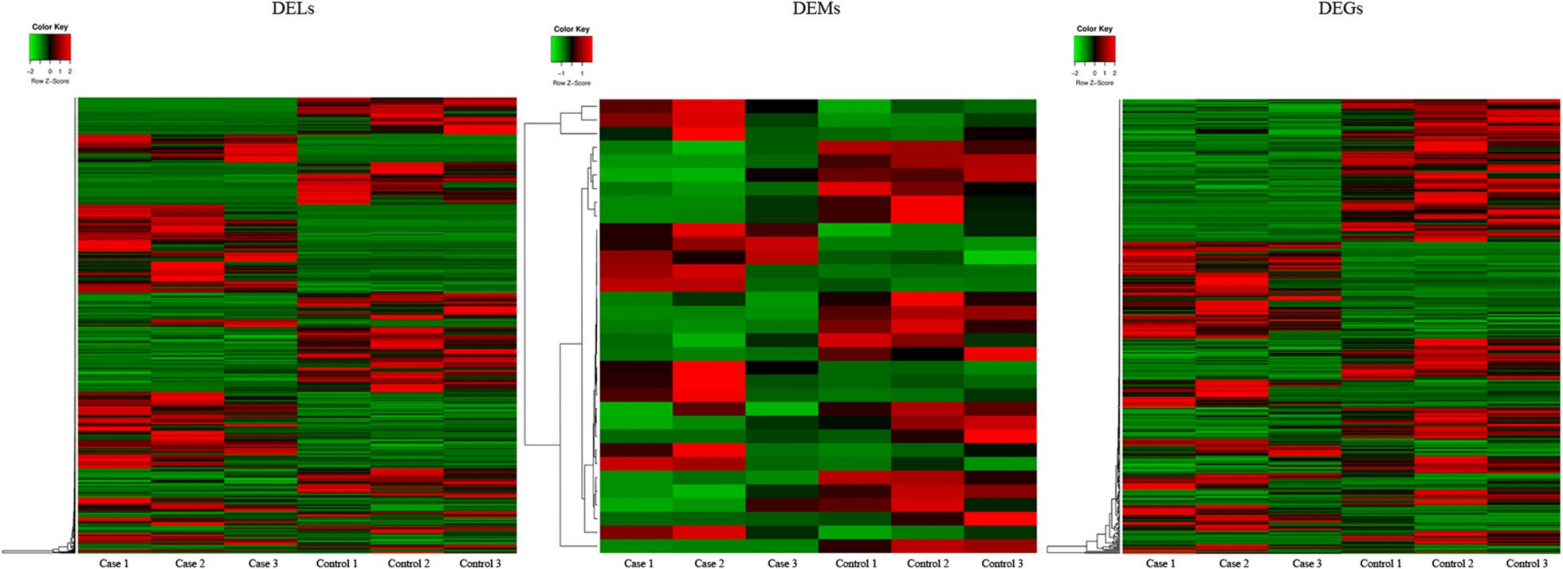
Heatmap analysis of DEL, DEM and DEGs. DEL: Differentially expressed lncRNAs; DEMs: Differentially expressed miRNAs; DEGs: Differentially expressed genes

According to the quality of the total RNA from 3 patients with RSA and 3 normal abortion personnel (Additional file 1: Figure S1), the transcriptome sequencing showed that 1008 DELs (523 upregulated and 485 downregulated), 475 DEGs (201 upregulated and 274 downregulated) and 37 DEMs (15 upregulated and 22 downregulated), which were shown in the heat map (Fig. 1) and the volcano map (Additional file 3: Figure S3, Additional file 4: Figure S4 and Additional file 5: Figure S5). Table 1 illustrated the DEGs and DELs (top 20), and Table 2 listed the top 15 DEMs. The thresholds of the screening data were *p* < 0.05 and |log_2_FC|> 1.

**Table 1:** Top 20 downregulated DELsand DEGs

Name	Log_2_FC	*p* value	FDR
*lncRNAs*			
lnc-LUC7L-2	− 12.39733625	2.73E−23	4.473E−19
lnc-SVIL-1	− 10.14199781	0.009439	0.8407162
lnc-GYPB-2	− 10.04160545	1.05E−14	6.891E−11
lnc-TAL1-3	− 9.098739111	1.69E−11	6.145E−08
lnc-GPAT4-4	− 8.301384128	2.41E−09	7.187E−06
lnc-CXorf58-2	− 8.001569916	0.040642	0.9999888
lnc-TAL1-2	− 7.993599712	1.46E−07	0.0003185
lnc-TAL1-1	− 7.473540526	5.28E−07	0.0010197
lnc-CCDC80-5	− 7.360578289	1.01E−14	6.891E−11
lnc-GPX2-4	− 7.17832599	2.87E−06	0.0047
lnc-GCDH-3	− 7.146089066	4.05E−06	0.0055418
lnc-ZNF674-14	− 6.715903208	5.78E−05	0.0364908
lnc-ANKRD34B-1	− 6.594566822	0.000117	0.065244
lnc-SLC4A1-1	− 6.591100728	3.72E−05	0.0259794
IL21R-AS1	− 6.574007081	0.000154	0.0803045
lnc-NT5C2-1	− 6.499432393	0.000265	0.1146595
lnc-LRRC71-4	− 6.493785307	0.001977	0.4445064
lnc-EEF1A1-1	− 6.4933232	0.000141	0.074564
lnc-MTA3-2	− 6.457648098	0.000217	0.098679
lnc-TBC1D2B-8	− 6.446282105	0.000182	0.0916568
*Gene*			
HBZ	− 12.29877063	1.1E−17	2.346E−14
HBE1	− 12.08390031	1.16E−08	6.391E−06
CTSE	− 10.90211575	2.04E−17	3.882E−14
PKLR	− 10.5396262	6.11E−15	6.549E−12
HBG1	− 10.25889722	9.69E−10	6.64E−07
AHSP	− 10.18762318	2.16E−15	2.614E−12
GYPB	− 9.839459815	6.36E−14	6.411E−11
RHAG	− 9.68168386	6.79E−13	6.123E−10
HBG2	− 9.436337355	6.3E−129	1.07E−124
TGIF2-RAB5IF	− 9.153251114	0.019158	0.674672
CD5L	− 8.968198622	4.78E−09	2.826E−06
GFI1B	− 8.740214677	1.55E−09	9.851E−07
KLF1	− 8.631727943	4.25E−10	3.164E−07
ABHD14A-ACY1	− 8.592860821	0.027891	0.7822296
TRIM10	− 8.290726755	4.07E−09	2.49E−06
SLC4A1	− 7.487911126	1.84E−73	1.575E−69
DUS4L-BCAP29	− 7.463007241	1.78E−15	2.352E−12
FAM83A	− 7.25200236	0.002489	0.2557698
GYPA	− 7.056613955	6.81E−14	6.484E−11
APOC3	− 6.933554968	0.00925	0.4944919

Log_2_FoldChange: Log_2_FC; DEL: differential expression LncRNAs; DEGs: differential expression genes

**Table 2:** Top 15 upregulated DEMs

Name	Log_2_FC	*p* value	FDR
hsa-let-7d-3p	2.3366134	0.0022868	0.1875138
chr19_19396	2.327217	0.0003966	0.4314759
hsa-miR-6715b-3p	2.2766042	0.0036217	0.2375866
hsa-miR-10b-5p	1.7264499	0.0004681	0.0658066
hsa-miR-210-5p	1.711749	0.0080521	0.4170136
hsa-miR-181c-5p	1.6300442	0.0079254	0.4170136
hsa-let-7b-5p	1.6297179	0.0015161	0.1421603
hsa-let-7i-5p	1.6187541	0.0008794	0.0979275
hsa-miR-187-3p	1.5738613	0.0015892	0.1421603
hsa-miR-653-5p	1.432427	0.0097668	0.4452522
hsa-let-7d-5p	1.3072349	0.0325617	0.9977985
hsa-miR-874-3p	1.2936117	0.0135001	0.5535043
hsa-miR-10b-3p	1.2624656	0.0379635	0.9977985
hsa-miR-146a-5p	1.1734703	0.0460092	0.9977985
hsa-miR-3690	1.1308818	0.045213	0.9977985

Log2FoldChange: Log2FC; DEMs: differential expression miRNAs

### GO and pathway analysis of DELs

**Fig. 2:**
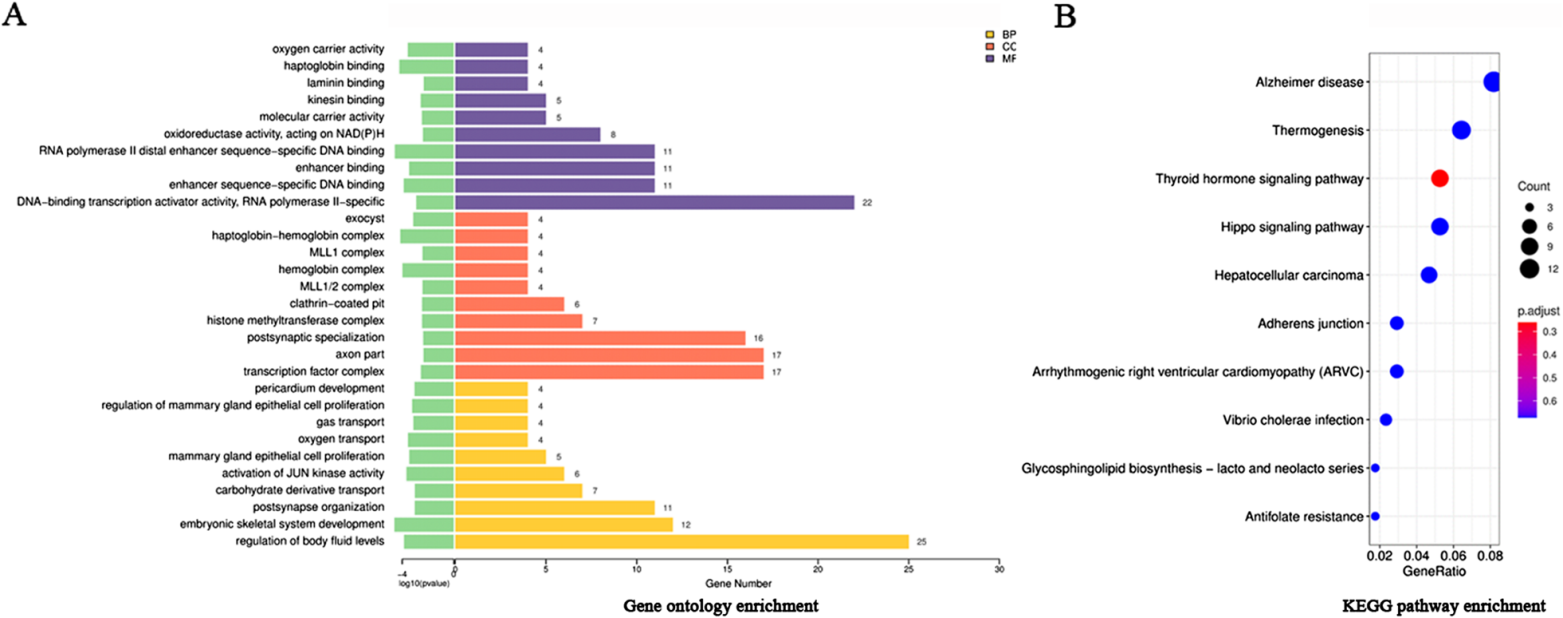
GO enrichment items and KEGG pathway analysis of DELs. **a** Showed that the top 10 enriched GO pathways of DELs were sorted by significance in biological process (BP), cellular component (CC) and molecular function (MF), respectively. **b** Showed the top 10 enriched KEGG pathways of DELs

To further study the transcriptome differences between the two groups, we performed GO and KEGG pathway analyses of DELs. In the Table 3 and Fig. 2a, the results of the top 10 enriched GO pathways of DELs showed that the biological process (BP) changes were in the regulation of body fluid levels, embryonic skeletal system development, postsynapse organization, carbohydrate derivative transport, activation of JUN kinase activity, mammary gland epithelial cell proliferation, oxygen transport, gas transport, regulation of mammary gland epithelial cell proliferation and pericardium development. Additionally, the cell component (CC) changes of DELs were obviously enriched in transcription factor complex, axon part, postsynaptic specialization, histone methyltransferase complex, clathrin-coated pit, MLL1/2 complex, hemoglobin complex, MLL1 complex, haptoglobin-hemoglobin complex and exocyst. Moreover, molecular function (MF) changes were mainly enriched in DNA-binding transcription activator activity, RNA polymerase II-specific, enhancer sequence-specific DNA binding, enhancer binding, RNA polymerase II distal enhancer sequence-specific DNA binding, oxidoreductase activity, acting on NAD(P)H, molecular carrier activity, kinesin binding, laminin binding, haptoglobin binding and oxygen carrier activity. As shown in the Table 4 and Fig. 2b, the top 10 enriched KEGG pathways of DELs were in Alzheimer’s disease, Thermogenesis, Thyroid hormone signaling pathway, Hippo signaling pathway, Hepatocellular carcinoma, Adherens junction, Arrhythmogenic right ventricular cardiomyopathy (ARVC), Vibrio cholerae infection, Glycosphingolipid biosynthesis—lacto and neolacto series and Antifolate resistance.

**Table 3:** Top 10 enriched GO pathways of DELs

Terms	Pathway description	Count	*p* value
*BP*			
GO:0050878	Regulation of body fluid levels	25	0.000139
GO:0048706	Embryonic skeletal system development	12	2.5E−05
GO:0099173	Postsynapse organization	11	0.000945
GO:1901264	Carbohydrate derivative transport	7	0.000968
GO:0007257	Activation of JUN kinase activity	6	0.000215
GO:0033598	Mammary gland epithelial cell proliferation	5	0.000357
GO:0015671	Oxygen transport	4	0.00028
GO:0015669	Gas transport	4	0.000741
GO:0033599	Regulation of mammary gland epithelial cell proliferation	4	0.000596
GO:0060039	Pericardium development	4	0.000911
*CC*			
GO:0005667	Transcription factor complex	17	0.002842
GO:0033267	Axon part	17	0.004541
GO:0099572	Postsynaptic specialization	16	0.004294
GO:0035097	Histone methyltransferase complex	7	0.003545
GO:0005905	Clathrin-coated pit	6	0.003517
GO:0044665	MLL1/2 complex	4	0.003743
GO:0005833	Hemoglobin complex	4	0.000105
GO:0071339	MLL1 complex	4	0.003743
GO:0031838	Haptoglobin-hemoglobin complex	4	7.15E−05
GO:0000145	Exocyst	4	0.000728
*MF*			
GO:0001228	DNA-binding transcription activator activity, RNA polymerase II-specific	22	0.001229
GO:0001158	Enhancer sequence-specific DNA binding	11	0.000132
GO:0035326	Enhancer binding	11	0.000351
GO:0000980	RNA polymerase II distal enhancer sequence-specific DNA binding	11	2.65E−05
GO:0016651	Oxidoreductase activity, acting on NAD(P)H	8	0.004119
GO:0140104	Molecular carrier activity	5	0.003458
GO:0019894	Kinesin binding	5	0.002799
GO:0043236	Laminin binding	4	0.004743
GO:0031720	Haptoglobin binding	4	6E−05
GO:0005344	Oxygen carrier activity	4	0.000265

BP: Biological process; CC: cellular component; MF: molecular function

**Table 4:** Top 10 enriched KEGG pathways of DELs

ID	Pathway description	Count	*p* value
hsa05010	Alzheimer disease	14	0.025553
hsa04714	Thermogenesis	11	0.010243
hsa04919	Thyroid hormone signaling pathway	9	0.0010683
hsa04390	Hippo signaling pathway	9	0.0062426
hsa05225	Hepatocellular carcinoma	8	0.0268635
hsa04520	Adherens junction	5	0.0173581
hsa05412	Arrhythmogenic right ventricular cardiomyopathy (ARVC)	5	0.0238119
hsa05110	Vibrio cholerae infection	4	0.0213693
hsa00601	Glycosphingolipid biosynthesis—lacto and neolacto series	3	0.0190689
hsa01523	Antifolate resistance	3	0.0275527

### GO and pathway analyses of DEGs

**Fig. 3:**
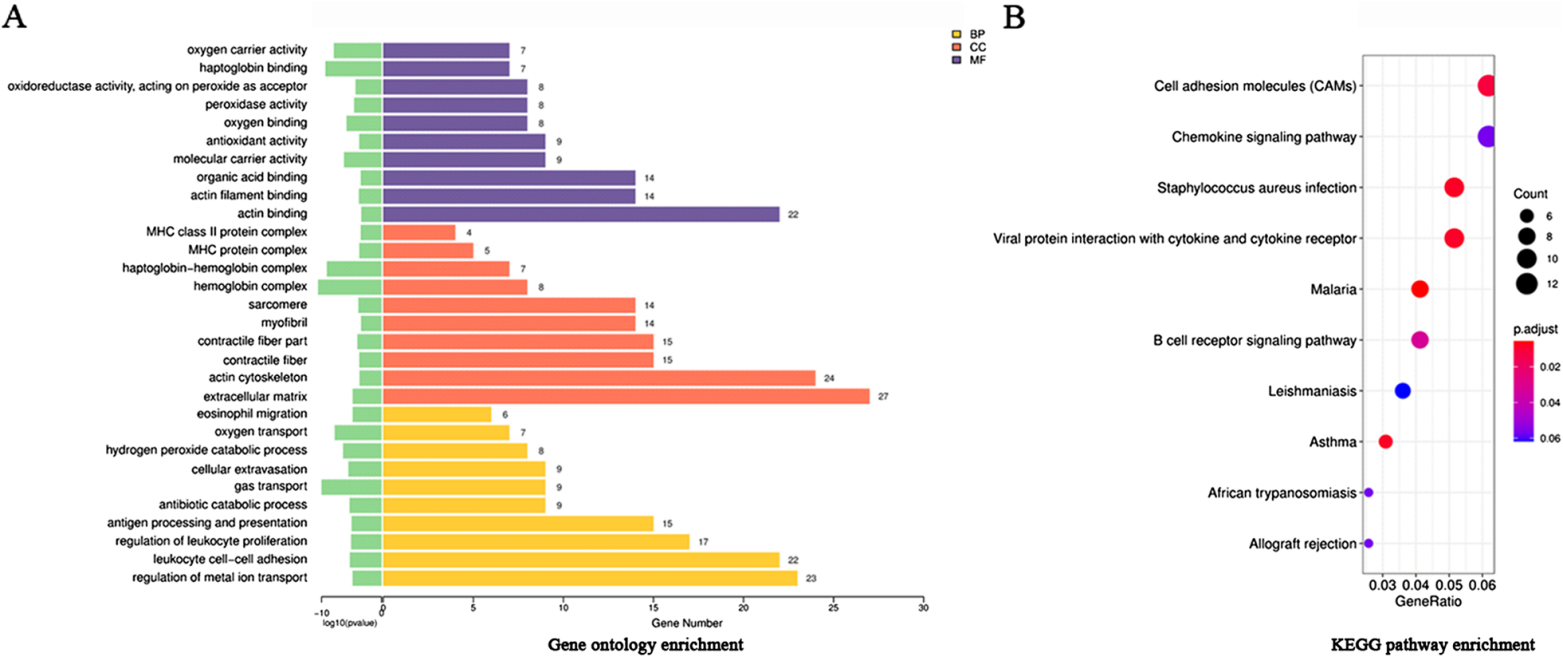
GO enrichment items and KEGG pathway analysis of DEGs. **a** Showed that the top 10 enriched GO pathways of DEGs were sorted by significance in biological process (BP), cellular component (CC) and molecular function (MF), respectively. **b** Showed the top 10 enriched KEGG pathways of DEGs

To further study the transcriptome differences between the two groups, we performed the GO and KEGG pathway analysis of DEGs. The results of the top 10 GO pathways of DEGs showed that changes in biological processes (BP) were mainly enriched in regulation of metal ion transport, leukocyte cell–cell adhesion, regulation of leukocyte proliferation, antigen processing and presentation, antibiotic catabolic process, gas transport, cellular extravasation, hydrogen peroxide catabolic process, oxygen transport and eosinophil migration. In addition, cell component (CC) changes were mainly concentrated in extracellular matrix, actin cytoskeleton, contractile fiber, contractile fiber part, myofibril, sarcomere, hemoglobin complex, haptoglobin-hemoglobin complex, MHC protein complex and MHC class II protein complex. Molecular function (MF) changes were mainly distributed in actin binding, actin filament binding, organic acid binding, molecular carrier activity, antioxidant activity, oxygen binding, peroxidase activity, oxidoreductase activity, acting on peroxide as acceptor, haptoglobin binding and oxygen carrier activity (Table 5 and Fig. 3a). The top 10 KEGG pathways of DEGs were mainly enriched in Cell adhesion molecules (CAMs), Chemokine signaling pathway, Staphylococcus aureus infection, Viral protein interaction with cytokine and cytokine receptor, Malaria, B cell receptor signaling pathway, Leishmaniasis, Asthma, African trypanosomiasis and Allograft rejection (Table 6 and Fig. 3b).

**Table 5:** Top 10 enriched GO terms of DEGs

Terms	Pathway description	Count	*p* value
*BP*			
GO:0010959	Regulation of metal ion transport	23	1.5E−05
GO:0007159	Leukocyte cell–cell adhesion	22	5.16E−06
GO:0070663	Regulation of leukocyte proliferation	17	8.10E−06
GO:0019882	Antigen processing and presentation	15	9.95E−06
GO:0017001	Antibiotic catabolic process	9	4.59E−06
GO:0015669	Gas transport	9	1E−10
GO:0045123	Cellular extravasation	9	2.88E−06
GO:0042744	Hydrogen peroxide catabolic process	8	3.94E−07
GO:0015671	Oxygen transport	7	1.5E−08
GO:0072677	Eosinophil migration	6	1.52E−05
*CC*			
GO:0031012	Extracellular matrix	27	1.57E−05
GO:0015629	Actin cytoskeleton	24	0.000225
GO:0043292	Contractile fiber	15	0.000198
GO:0044449	Contractile fiber part	15	9.38E−05
GO:0030016	Myofibril	14	0.000392
GO:0030017	Sarcomere	14	0.000138
GO:0005833	Hemoglobin complex	8	1.1E−08
GO:0031838	Haptoglobin-hemoglobin complex	7	7.81E−10
GO:0042611	MHC protein complex	5	0.000195
GO:0042613	MHC class II protein complex	4	0.000354
*MF*			
GO:0003779	Actin binding	22	0.000437
GO:0051015	Actin filament binding	14	0.000169
GO:0043177	Organic acid binding	14	0.000351
GO:0140104	Molecular carrier activity	9	5.64E−07
GO:0016209	antioxidant activity	9	0.000189
GO:0019825	Oxygen binding	8	1.49E−06
GO:0004601	Peroxidase activity	8	2.67E−05
GO:0016684	Oxidoreductase activity, acting on peroxide as acceptor	8	4.65E−05
GO:0031720	Haptoglobin binding	7	4.3E−10
GO:0005344	Oxygen carrier activity	7	1.13E−08

BP: Biological process; CC: cellular component; MF: molecular function

**Table 6:** Top 10 enriched KEGG pathways of DEGs

ID	Pathway description	Count	*p* value
hsa04514	Cell adhesion molecules (CAMs)	12	0.00023
hsa04062	Chemokine signaling pathway	12	0.00204
hsa05150	Staphylococcus aureus infection	10	1E−04
hsa04061	Viral protein interaction with cytokine and cytokine receptor	10	0.00014
hsa05144	Malaria	8	2.3E−05
hsa04662	B cell receptor signaling pathway	8	0.00078
hsa05140	Leishmaniasis	7	0.00248
hsa05310	Asthma	6	8.2E−05
hsa05143	African trypanosomiasis	5	0.00182
hsa05330	Allograft rejection	5	0.00205

### Construction of the lncRNA-miRNA-mRNA ceRNA network

**Fig. 4:**
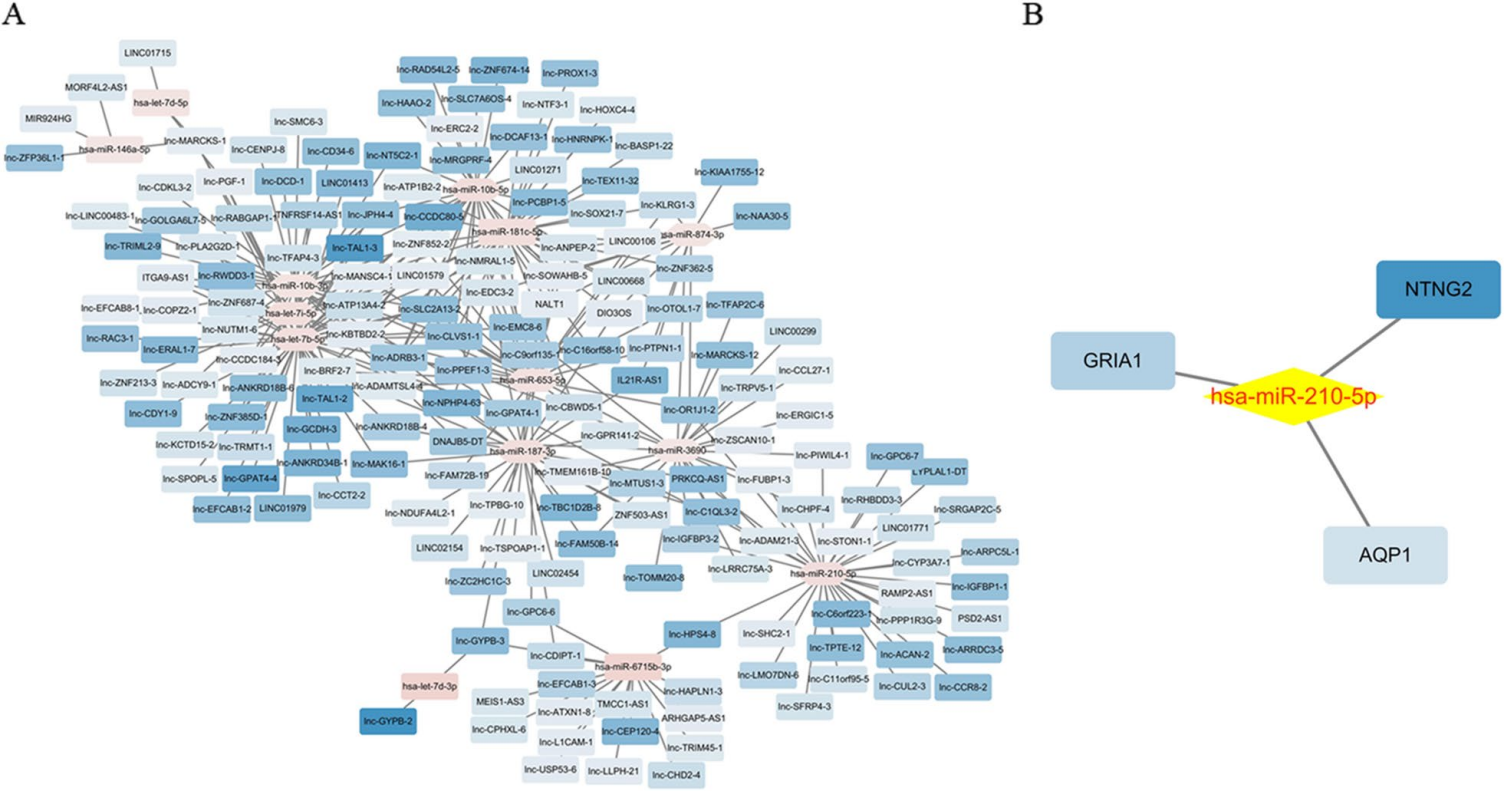
The interactions between lncRNA-miRNA and miRNA-genes were determined, respectively. **a** Showed the relationship between downregulated lncRNAs and upregulated miRNAs. **b** Listed the interactions between hsa-miR-210-5p and 3 genes

**Fig. 5:**
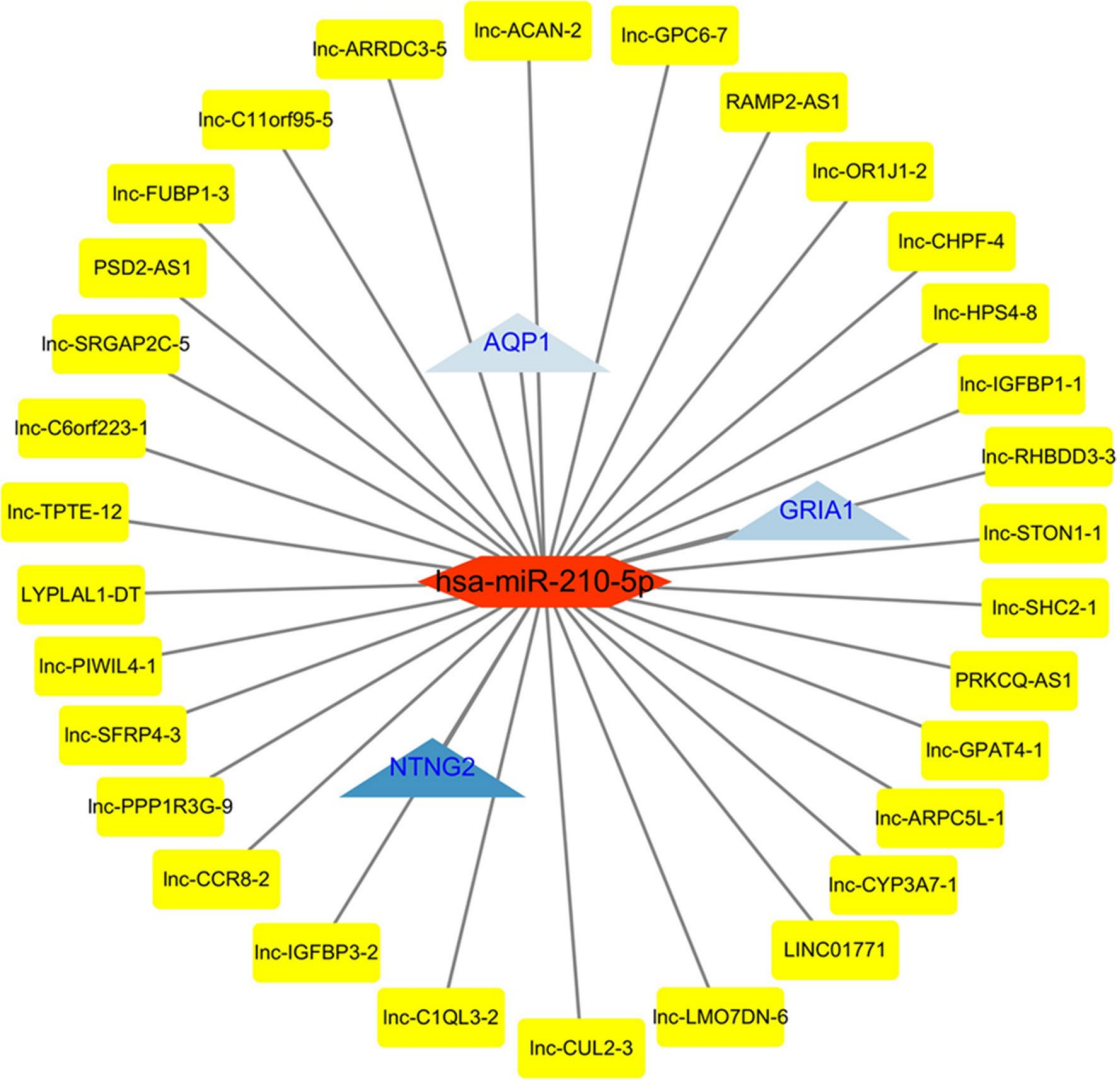
The ceRNA network was constructed between DELs, DEMs and DEGs

First, we constructed a lncRNA-miRNA network and miRNA-mRNA network. The lncRNA-miRNA network included 4607 negative interactions (1945 downregulated lncRNAs-upregulated miRNAs and 2662 upregulated lncRNAs- downregulated miRNAs), and the miRNA-mRNA network included 15 negative interactions (6 downregulated miRNAs- upregulated mRNAs and 9 upregulated miRNAs-downregulated mRNAs). Then, we constructed the lncRNA-miRNA-mRNA ceRNA network to identify their relationships based on the lncRNA, miRNA, and mRNA expression profiles, and plotted them using Cytoscape version 3.6.1. First, based on the threshold values (r < 0 and *p* value < 0.05), we evaluated the relationship between downregulated lncRNAs and upregulated miRNAs shown in Fig. 4a, and the relationship between upregulated lncRNAs and downregulated miRNAs was displayed in Additional file 2: Figure S2. Additionally, the results of the miRNA-mRNA relationship showed a significant link between hsa-miR-210-5p and mRNAs (NTNG2, GRIA1 and AQP1), as shown in Fig. 4b. Besides, we constructed the ceRNA network between DELs, DEMs and DEGs by the Pearson correlation coefficient. Finally, the ceRNA network contained 31 lncRNAs (PSD2-AS1, lnc-ACAN-2, lnc-STON1-1, lnc-HPS4-8, lnc-SHC2-1, lnc-LMO7DN-6, lnc-TPTE-12, lnc-ARRDC3-5, lnc-CHPF-4, lnc-OR1J1-2, lnc-GPAT4-1, lnc-ARPC5L-1, LYPLAL1-DT, lnc-PIWIL4-1, lnc-CCR8-2, lnc-RHBDD3-3, lnc-PPP1R3G-9, RAMP2-AS1, LINC01771, lnc-SFRP4-3, lnc-C1QL3-2, lnc-C6orf223-1, lnc-IGFBP3-2, lnc-CUL2-3, lnc-SRGAP2C-5, PRKCQ-AS1, lnc-C11orf95-5, lnc-IGFBP1-1, lnc-CYP3A7-1, lnc-GPC6-7 and lnc-FUBP1-3), 1 miRNA (hsa-miR-210-5p) and 3 mRNAs (NTNG2, GRIA1 and AQP1) as displayed in Fig. 5 and Table 7 (top20), illustrating that these molecules may be involved in the development of RSA.

**Table 7:** Construction of ceRNA network (Top 20)

lncRNA	lncRNA_miRNA cor	*p* value	miRNA	miRNA_mRNA cor	*p* value	mRNA	mRNA_lncRNA cor	*p* value
PSD2-AS1	-12.39733625	0.01666667	hsa-miR-210-5p	− 0.9428571	0.01666667	GRIA1	0.8857143	0.03333333
PSD2-AS1	-10.14199781	0.01666667	hsa-miR-210-5p	− 0.8857143	0.03333333	AQP1	0.9428571	0.01666667
PSD2-AS1	-10.04160545	0.01666667	hsa-miR-210-5p	− 0.8804063	0.02059873	NTNG2	0.9411239	0.005097541
lnc-ACAN-2	-9.098739111	0.03410942	hsa-miR-210-5p	− 0.9428571	0.01666667	GRIA1	0.8451543	0.03410942
lnc-ACAN-2	-8.301384128	0.03410942	hsa-miR-210-5p	− 0.8857143	0.03333333	AQP1	0.7775419	0.06872694
lnc-ACAN-2	-8.001569916	0.03410942	hsa-miR-210-5p	− 0.8804063	0.02059873	NTNG2	0.8261844	0.04269215
lnc-STON1-1	-7.993599712	0.03333333	hsa-miR-210-5p	− 0.9428571	0.01666667	GRIA1	0.7714286	0.1027778
lnc-STON1-1	-7.473540526	0.03333333	hsa-miR-210-5p	− 0.8857143	0.03333333	AQP1	1	0.002777778
lnc-STON1-1	-7.360578289	0.03333333	hsa-miR-210-5p	− 0.8804063	0.02059873	NTNG2	0.9411239	0.005097541
lnc-HPS4-8	-7.17832599	0.02059873	hsa-miR-210-5p	− 0.9428571	0.01666667	GRIA1	0.8804063	0.02059873

cor: Correlation

*p* value < 0.05 and cor ≤ −0.8 indicate that there is a negative correlation

## Discussion

Recurrent spontaneous abortion is one of the common complications of pregnancy. In the past few decades, the disease has caused heavy psychological burden for couples who want to have children and their families. However, due to the high cost of treatment, many families have failed to realize their desire to have children. The present study showed that we found 1008 DELs, 475 DEGs and 37 DEMs in 3 patients with RSA and 3 normal abortion personnel by transcriptome sequencing of villous tissue samples. We also constructed a novel lncRNA-related ceRNA network containing 31 lncRNAs, 1 miRNA (hsa-miR-210-5p) and 3 mRNAs (NTNG2, GRIA1 and AQP1). The results may provide a theoretical basis for elucidating the mechanism of RSA.

*NTNG2* (*Netrin G2*) the position of which on chromosome is 9q34.13 and encodes the protein NTNG2, a membrane anchor protein. It was found to promote the growth of axons and dendrites. Studies on the correlation of gene polymorphisms in schizophrenia revealed that *NTNG1* and its paralogues for *NTNG2* gene may be related to the pathophysiology of schizophrenia [13–15]. Another paper reported by Maroofian et al. illustrated that NTNG2 played a key role in neurotypical development [16]. Therefore, we speculate that NTNG2 and NTNG1 may play a role in neurological disorders. In addition, based on the bioinformatics analysis of the pediatric onset of multiple sclerosis, genes such as *NTNG2* were found to be nodes of the network, and the expression of some miRNAs were significantly correlated with brain volume [17]. But to date, there is no report on the NTNG2-associated network in RSA.

*GRIA1* (*Glutamate Ionotropic Receptor AMPA Type Subunit 1*) is located on the chromosome 5q33.2 and the encoded protein is the main excitatory neurotransmitter receptor in the mammalian brain. It is reported to be a ligand-gated ion channel, regulating the secretion of follicle-stimulating hormone and luteinizing hormone by controlling gonadotropin releasing hormone. Recently, Sugimoto et al. discovered that the gene was linked to the ovulation rate in cattle [18]. Cushman et al. found the correlation between *GRIA1* SNPs and cattle infertility [19]. In addition, Sheikhha et al. also observed the relationship between *GRIA1* variants and ovarian response to human menopausal gonadotropin in the group of Iranian women [20]. The above studies show that GRIA1 plays an important role in diseases related to pregnancy in women. But so far, the role of GRIA1 in RSA has not been reported. In our study, we used Cytoscape software to construct a network to combine noncoding RNAs to explore its function in RSA.

*AQP1* (*Aquaporin 1*), located on chromosome 7p14.3, contains 4 exons. Some reports have revealed that AQP1 plays an important role in acute lung injury caused by endotoxic shock, delaying the occurrence of renal cyst, and acute lung and brain injury [21, 22]. Su et al. used lipopolysaccharide (LPS)-induced murine model of acute lung injury to detect the function of AQP1, suggesting that AQP1 may be involved in the progression of acute lung injury [23]. Also, noncoding RNAs interacting with AQP1, were involved in the development of acute lung injury. Long noncoding RNA CASC2 can reduce the apoptosis of lung epithelial cells and improve acute lung injury by regulating the miR-144-3p/AQP1 axis [24]. Recent studies have shown that AQP1 participated in the occurrence of diseases through the ceRNA network [25, 26]. Tang et al. observed that lncRNA CASC2 acted as miR-144-3p, and directly interacted with AQP1 after LPS induced A549 cells [25]. After lipopolysaccharide (LPS) induced sepsis, Fang et al. found that *AQP1* has been reported to competitively bind to lncRNA H19 and regulated the expression of miRNA-874 [26]. But so far, there has been no study on lncRNA-miRNA-AQP1 in RSA.

At present, some researchers have obtained some results about recurrent abortion through transcriptome sequencing [27, 28]. In this study, we firstly performed transcriptome sequencing analysis on the tissues of 3 patients with RSA and 3 patients with normal abortion, and found key molecules by constructing lncRNA-related ceRNA network, which is helpful to explore the pathogenic mechanism of RSA. However, there are some limitations: (1) the sample size was insufficient; (2) the lncRNAs-miRNAs linked with RSA were not verified; (3) the lncRNA-mediated ceRNA network in RSA was not verified. In the future, we will continue to collect a large number of samples for verification, and further analyze the ceRNA network in RSA by transcriptome analyses, and use molecular biology to verify this network, providing a theoretical basis for the elucidation and treatment of RSA.

## Conclusion

In summary, a lncRNA-related ceRNA network containing 31 lncRNAs, 1 miRNA (hsa-miR-210-5p) and 3 mRNAs (NTNG2, GRIA1 and AQP1) was constructed. The results may provide the basic theory for elucidating the mechanism underlying RSA.

## Supplementary Information

**Additional file 1**. **Figure S1.** The quality of the total RNA from 3 patients with RSA and 3 normal abortion patients

**Additional file 2**. **Figure S2.** The relationship between upregulated lncRNAs and downregulated miRNAs

**Additional file 3**. **Figure S3.** Volcano map of the differentially expressed lncRNAs

**Additional file 4**. **Figure S4.** Volcano map of the differentially expressed mRNAs

**Additional file 5**. **Figure S5.** Volcano map of the differentially expressed miRNAs. A represents the known miRNAs; B represents the novel miRNAs

## Data Availability

The datasets generated and/or analysed during the current study are deposited in Genome Sequence Archive (GSA) for Human under analysis accession number "HRA001475" and "HRA001505".

## Abbreviations

RSA: Recurrent spontaneous abortion
DELs: Differentially expressed lncRNAs
DEMs: Differentially expressed miRNAs
DEGs: Differentially expressed genes
lncRNAs: Long non coding RNAs
GO: Geno ontology
KEGG: Kyoto encyclopedia of genes and genomes
BP: Biological process
CC: Cell composition
MF: Molecular function
CAMs: Cell adhesion molecules
NTNG2: Netrin G2
GRIA1: Glutamate Ionotropic Receptor AMPA Type Subunit 1
AQP1: Aquaporin 1
LPS: Lipopolysaccharide
