# Supplementary figures and images for: Transcriptome sequencing identified the ceRNA network associated with recurrent spontaneous abortion
Source: BMC Med Genomics. 2021 Nov 23;14:278. doi: 10.1186/s12920-021-01125-4 (PMC8609870; doi:10.1186/s12920-021-01125-4)

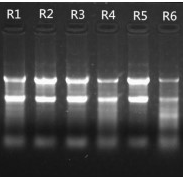

Supplement: Supplementary file 1 — Additional file 1. Figure S1. The quality of the total RNA from 3 patients with RSA and 3 normal abortion patients [file 12920_2021_1125_MOESM1_ESM.tif]

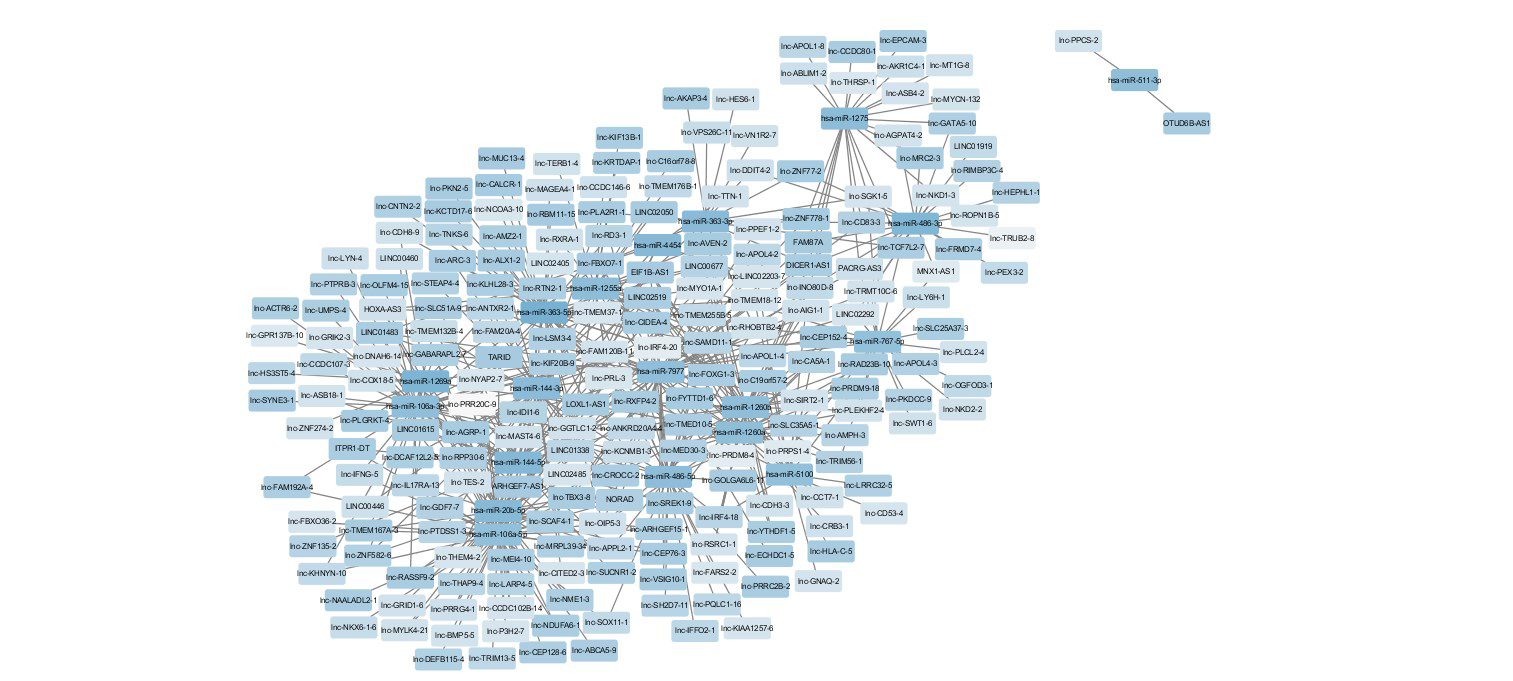

Supplement: Supplementary file 2 — Additional file 2. Figure S2. The relationship between upregulated lncRNAs and downregulated miRNAs [file 12920_2021_1125_MOESM2_ESM.tif]

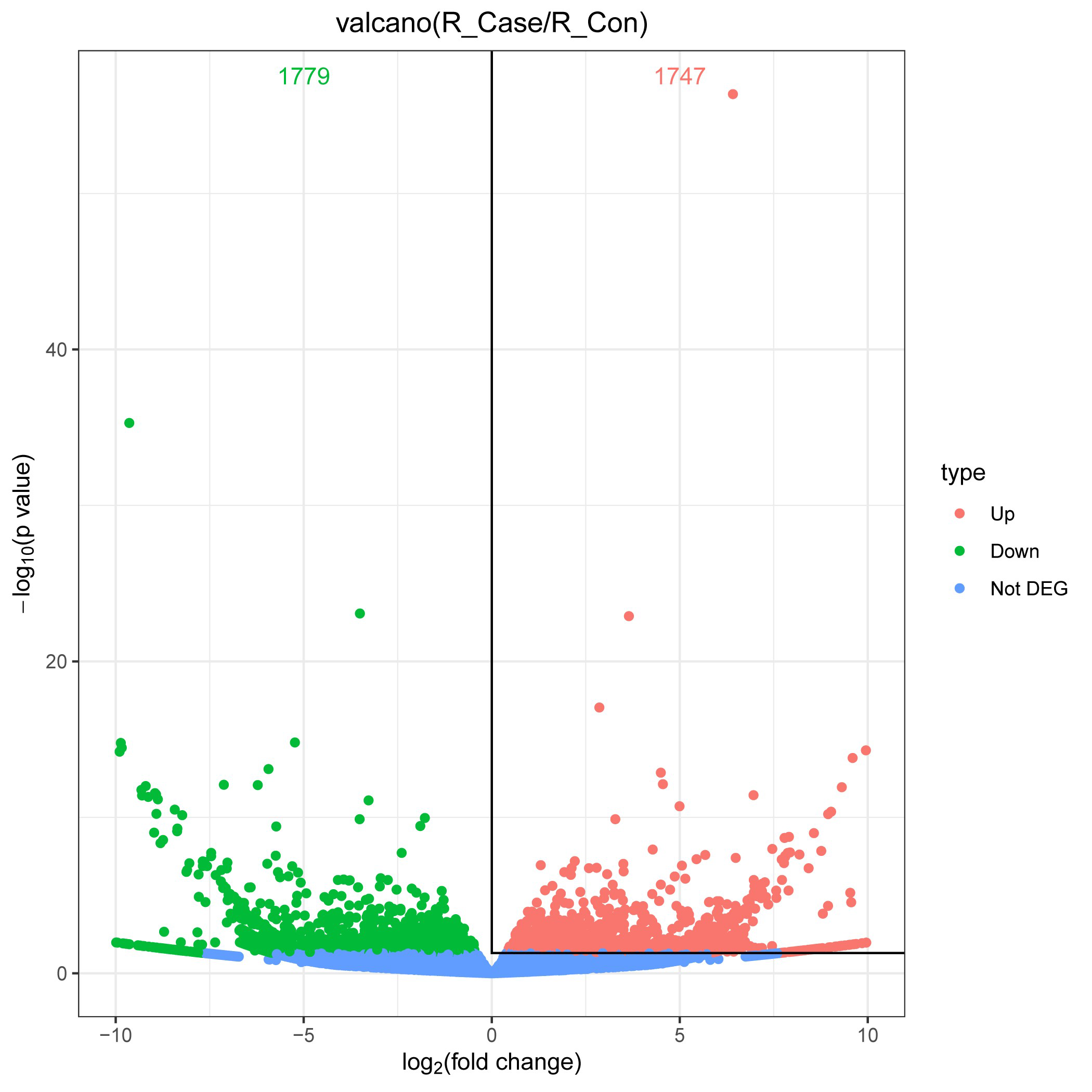

Supplement: Supplementary file 3 — Additional file 3. Figure S3. Volcano map of the differentially expressed lncRNAs [file 12920_2021_1125_MOESM3_ESM.tif]

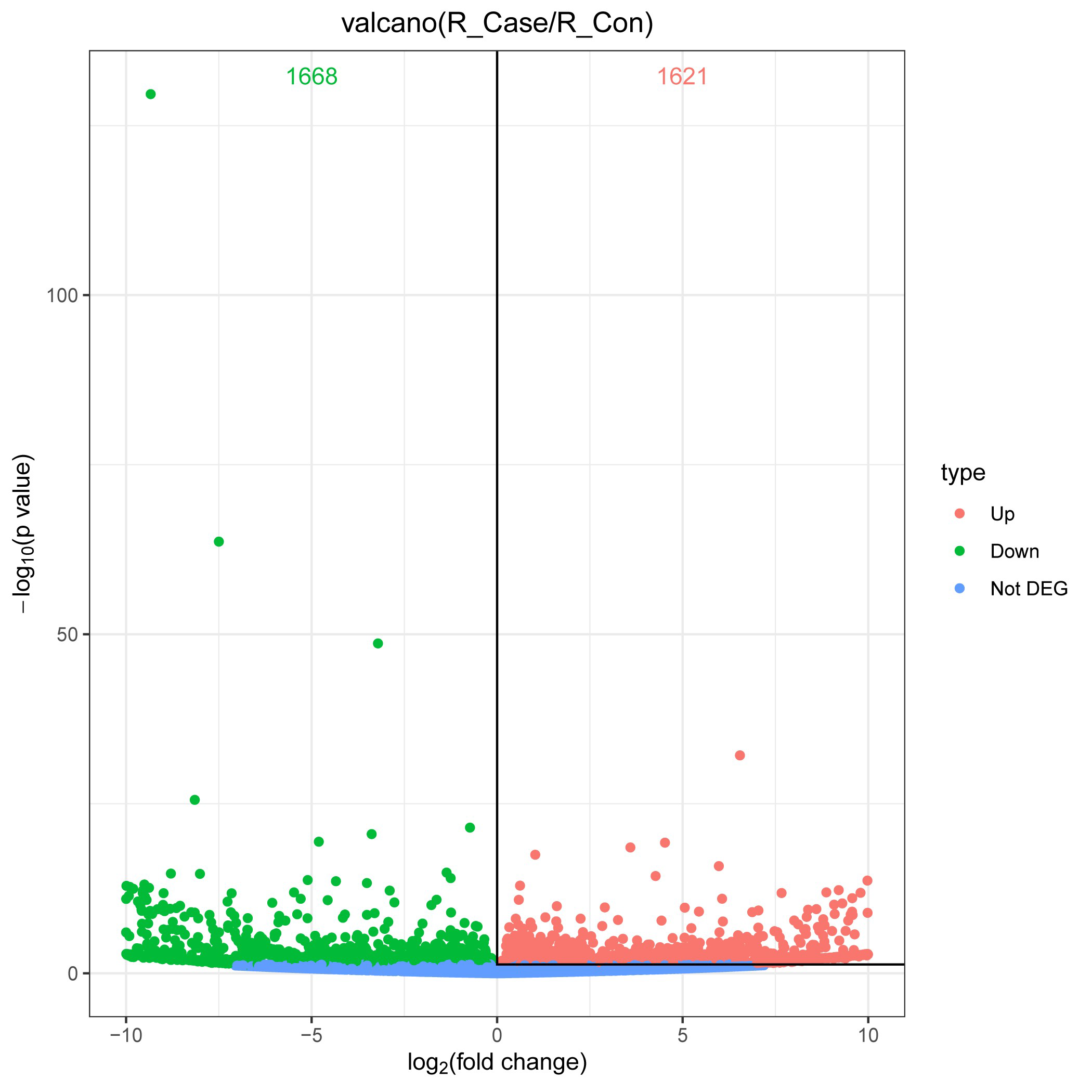

Supplement: Supplementary file 4 — Additional file 4. Figure S4. Volcano map of the differentially expressed mRNAs [file 12920_2021_1125_MOESM4_ESM.tif]

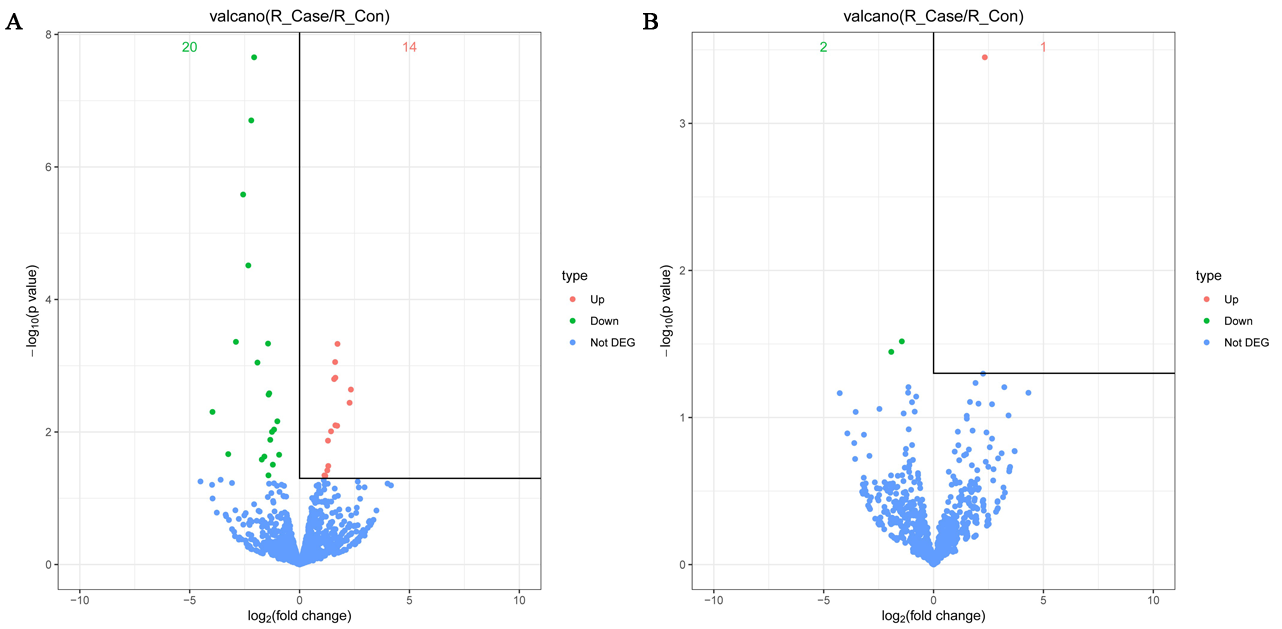

Supplement: Supplementary file 5 — Additional file 5. Figure S5. Volcano map of the differentially expressed miRNAs. A represents the known miRNAs; B represents the novel miRNAs [file 12920_2021_1125_MOESM5_ESM.tif]
